# Extensive genetic diversity and rapid population differentiation during blooms of *Alexandrium fundyense* (Dinophyceae) in an isolated salt pond on Cape Cod, MA, USA

**DOI:** 10.1002/ece3.373

**Published:** 2012-09-13

**Authors:** Mindy L Richlen, Deana L Erdner, Linda A R McCauley, Katie Libera, Donald M Anderson

**Affiliations:** 1 Woods Hole Oceanographic InstitutionWoods Hole, Massachusetts, 02543; 2 Marine Science Institute, University of Texas at AustinPort Aransas, Texas, 78373

**Keywords:** *Alexandrium*, *Amoebophrya*, dinoflagellate, Gulf of Maine, microsatellites, Nauset Marsh, paralytic shellfish poisoning.

## Abstract

In Massachusetts, paralytic shellfish poisoning (PSP) is annually recurrent along the coastline, including within several small embayments on Cape Cod. One such system, the Nauset Marsh System (NMS), supports extensive marshes and a thriving shellfishing industry. Over the last decade, PSP in the NMS has grown significantly worse; however, the origins and dynamics of the toxic *Alexandrium fundyense* (Balech) populations that bloom within the NMS are not well known. This study examined a collection of 412 strains isolated from the NMS and the Gulf of Maine (GOM) in 2006–2007 to investigate the genetic characteristics of localized blooms and assess connectivity with coastal populations. Comparisons of genetic differentiation showed that *A. fundyense* blooms in the NMS exhibited extensive clonal diversity and were genetically distinct from populations in the GOM. In both project years, genetic differentiation was observed among temporal samples collected from the NMS, sometimes occurring on the order of approximately 7 days. The underlying reasons for temporal differentiation are unknown, but may be due, in part, to life-cycle characteristics unique to the populations in shallow embayments, or possibly driven by selection from parasitism and zooplankton grazing; these results highlight the need to investigate the role of selective forces in the genetic dynamics of bloom populations. The small geographic scale and limited connectivity of NMS salt ponds provide a novel system for investigating regulators of blooms, as well as the influence of selective forces on population structure, all of which are otherwise difficult or impossible to study in the adjacent open-coastal waters or within larger estuaries.

## Introduction

**Figure 1:**
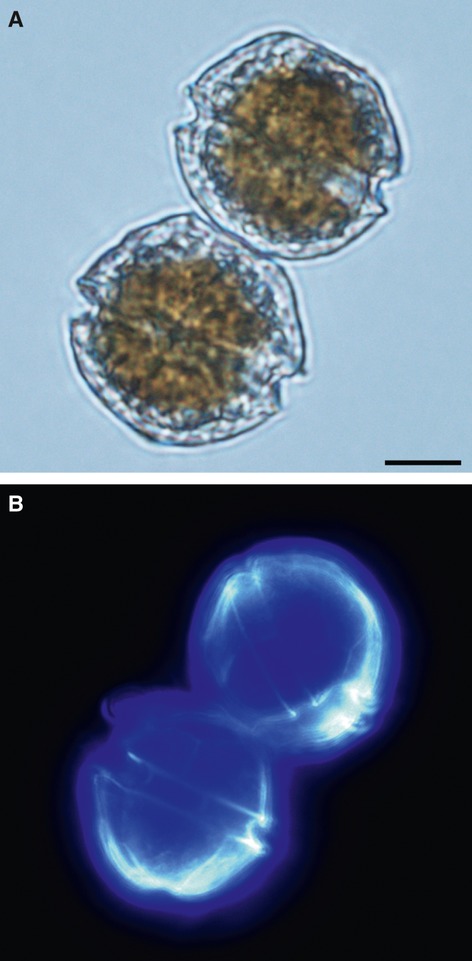
*Alexandrium fundyense* micrographs taken using (A) light microscopy (LM), (B) epifluorescence, showing chlorophyll autofluorescence, (C) LM, chain of two cells, (D) epifluorescence, calcofluor-stained cells. Scale bar = 10 μm.

The harmful algal bloom (HAB) syndrome known as Paralytic Shellfish Poisoning (PSP) is a significant and growing problem worldwide, with negative and sometimes devastating impacts on human consumers of contaminated seafood and local economies dependent on the shellfish industry. Paralytic Shellfish Poisoning outbreaks have also been linked to seabird and marine mammal mortalities, thus demonstrating the potential for serious ecosystem impacts (Anderson and White 1992; Shumway et al. 2003). Dinoflagellates within the genus *Alexandrium*, and in particular within the “tamarense” (*A. tamarense*, *A. fundyense*, and *A. catenella*) species complex (Fig 1), are responsible for many of these PSP outbreaks (Cembella 1998). In the US, PSP is prevalent along much of the west coast and more recently has also emerged as a major HAB problem in the northeastern Atlantic, particularly in the Gulf of Maine (GOM). Annual blooms of *A. fundyense*^1^ now impact most coastal areas of the northeastern United States and Canada, prompting the establishment of shellfish monitoring programs throughout the region.

In Massachusetts, PSP is annually recurrent along the coastline, including within a number of small embayments and salt ponds on Cape Cod. These embayments are characterized by restricted circulation and limited tidal flushing, receiving freshwater from groundwater flow and saltwater through one or more inlets to the sea. One such system on outer Cape Cod, the Nauset Marsh System (NMS), supports extensive marshes and thriving commercial and recreational shellfishing industries. This economically and socially important system also has a long history of PSP outbreaks associated with annual episodes of *Alexandrium* blooms, which are initiated and most intense in the landward extensions of the estuary.

Over the last several decades, the PSP problem in the NMS has worsened significantly, both in terms of intensity and duration. Anecdotal reports also suggest that PSP is occurring earlier and lasting longer than in the past. There are several explanations for these trends, including the increased retention and proliferation of toxic cells and dormant resting cysts in areas that are not well flushed in the system, as well as population stimulation by eutrophication pressures linked to the rapid development of the watershed through the years. Additionally, two major storms that occurred in 1991 may have altered flushing patterns and dispersed cysts within the NMS, thereby augmenting the magnitude of subsequent *A. fundyense* blooms.

Outbreaks in southern New England (i.e., south of Massachusetts Bay) are generally confined to salt ponds and embayments, suggestive of “point sources” of cells originating from locally germinated cysts (Anderson et al. 1983; Anderson and Stolzenbach 1985; Hattenrath et al. 2010). Although salt ponds in the NMS show strong characteristics of point-source *A. fundyense* habitats, it is clear that this estuary is in a transition area between the localized, estuarine populations of southern New England and the widespread, open-coastal populations of the GOM (e.g., Anderson et al. 2005a); thus, the system can thus potentially receive cells, and/or export them into the coastal flow. The implication is that the NMS may contain populations that originate *in situ*, as well as populations that have been introduced through Nauset Inlet from adjacent coastal waters during times of flood tide and downwelling-favorable winds. This exchange may also occur in the opposite direction as well – populations may be advected out of the system, into the coastal flow, thus mixing with southern GOM populations and affecting downstream shellfish resources.

This study investigated the genetic composition of *Alexandrium* blooms in the NMS to elucidate genetic linkages with coastal populations and to help determine the extent to which *Alexandrium* blooms within the salt ponds of the NMS are initiated solely within the system, and the degree to which cells from blooms are transported in and out of the NMS. In addition to examining the degree of genetic differentiation and connectivity between bloom populations in NMS and the GOM, we also hoped to gain insight into the potential effects of restricted geography on the genetic composition and diversity of populations within shallow, isolated embayments. Due to the extremely small geographic scale and limited connectivity with coastal populations in the GOM, this estuary provides a novel system for studying the genetic diversity and dynamics of *Alexandrium* blooms, as they appear to be largely restricted from genetic exchange with regional populations. These characteristics also make this study site an effective “natural laboratory” for the investigation of many important aspects of *Alexandrium* autoecology affecting bloom development and termination, such as life-cycle events as well as the influence of selective forces on population dynamics, all of which are otherwise difficult or impossible to study in the adjacent open-coastal waters, larger estuarine systems, or mesocosms.

To assess fine-scale spatial and temporal genetic diversity of *A. fundyense* populations, we employed rapidly evolving microsatellite markers. The ability of these markers to detect fine-scale variability among and sometimes within populations has provided researchers with an effective tool for exploring a diversity of questions regarding the population dynamics and molecular ecology of phytoplankton, at a resolution impossible to achieve with DNA sequencing. Microsatellites have been used in investigations of genetic differentiation across global and broad regional scales (Nagai et al. 2007, 2009; McCauley et al. 2009), to examine the genetic diversity and structure of bloom populations (Evans et al. 2005; Rynearson and Armbrust 2005; Rynearson et al. 2006) including the potential detection of cryptic species (Adams et al. 2009), infer the geographic origins of populations (Masseret et al. 2009), and to explore questions regarding life-cycle effects and phenotypic variability on population genetic structure (Iglesias-Rodriguez et al. 2006; Alpermann et al. 2009). Here, we used microsatellite markers to examine the genetic diversity and population structure of *Alexandrium* blooms that appear to be largely localized within the NMS. The study objectives were to: (1) characterize potential linkages among *Alexandrium* blooms within the NMS and with those in the wider GOM; (2) examine the effects of geographic restriction on the genetic diversity and population structure of cells within Salt Pond, where bloom residence times are high; and (3) examine population composition throughout a natural bloom.

## Methods

### Study site

**Figure 2:**
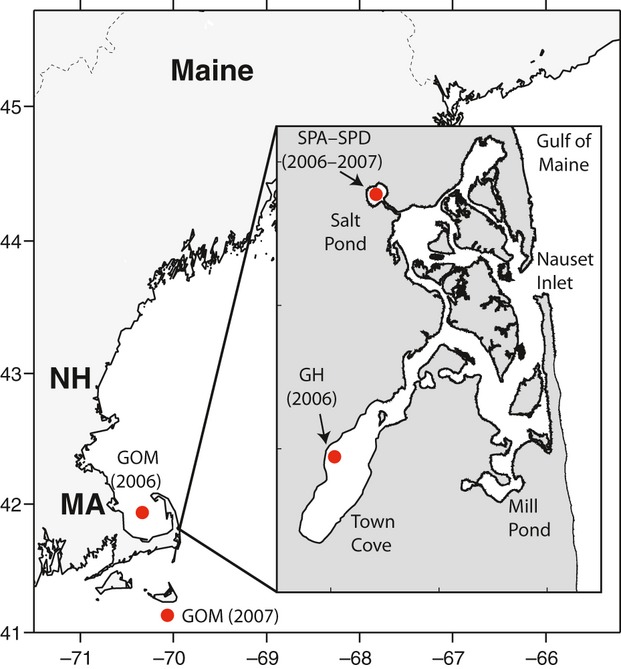
Map of the Nauset Marsh System and the Gulf of Maine showing location of field site for samples collected in 2006–2007. Sample collection year is indicated after station label. Star symbol indicates sample collection site.

The NMS is a coastal embayment of the Cape Cod National Seashore and is protected from the Atlantic by a rapidly changing barrier beach with a single connection through Nauset inlet (Fig 2). Its extensive marshes support thriving commercial and recreational shellfishing industries where quahogs, soft-shell clams, and razor clams are harvested along nearly all stretches of the shoreline and tidal flats. Terminal kettle ponds within the NMS include Salt Pond, Mill Pond, and Town Cove. Salt Pond is a circular, shallow embayment, approximately 82,200 m^2^ in area, with average and maximum depths of approximately 3.4 and 7 m, respectively, at slack low tide. There are no stream or river inputs of freshwater, but groundwater does enter the pond via springs, which keeps the pond more brackish than the incoming tidal flow.

Freshwater flux to Salt Pond and the shallow inlet channel (0.5 km long, 30 m wide, 0.5 m deep at low tide) allows tidal exchange with the wider marsh system that connects to the ocean at Nauset inlet. Tidal mixing between Salt Pond and the NMS is efficient, equivalent to a maximum potential flushing rate of 0.4–0.7 day^−1^, and is primarily due to an estuarine-like circulation in which flood-tide waters sink and vertically displace less dense pond water, which is then removed with the subsequent ebb tide (Anderson and Stolzenbach 1985).

### Sampling

Phytoplankton samples were collected from near surface waters of Salt Pond and Goose Hummock in the NMS during the bloom seasons of 2006–2007, and from Massachusetts Bay (2006) and Nantucket Shoals (2007) in the GOM (Fig 2). Single *Alexandrium* cells (contained in >20 and <64 μm Nitex-sieved field samples) were manually isolated via micropipette into the wells of a flat bottom 96-well plate containing 250 μL of f/2 -Si medium (Guillard 1975; Anderson et al. 1994). All cultures were maintained at 15 or 20°C at 250 μmol photons m^−2^ s^−1^ on a 14:10 light:dark (L:D) cycle. A total of 11 samples were collected during the course of the study: six in 2006 and five in 2007 (Table 1). In 2006, samples were collected from Salt Pond (herein termed 06SPA-06SPD), Goose Hummock (06GH), and Massachusetts Bay (06GOM). The 2007 sample set comprised four Salt Pond populations (07SPA-07SPD) and one from Nantucket Shoals (07GOM).

**Table 1:** Information regarding the sampling date, microsatellite loci, allelic characteristics, and clonal diversity of *Alexandrium fundyense* populations from Salt Pond, Cape Cod, MA, USA. Data are shown for each population sampled, and combined datasets for each project year. *N* = number of individuals genotyped, G:N – clonal diversity (Ellstrand and Roose 1987), and probabilities of identity (*P*_(ID)_ and *P*_(ID)sib_) across all loci

			Number of alleles				Size range of alleles						
							
Population ID	Sampling date	N	At15	At23	At27	At39	At15	At23	At27	At39	G:N	*P*_(ID)_	*P*_(ID)sib_
06SPA	4/26/06	29	11	8	5	7	240–266	176–204	160–168	136–150	0.97	3.26E-05	2.72E-02
06SPB	5/03/06	35	6	11	5	5	240–250	176–204	160–168	132–142	0.86	9.27E-05	3.35E-02
06SPC	5/10/06	30	7	8	5	3	238–266	176–202	160–168	138–142	0.90	1.14E-04	3.48E-02
06SPD	5/17/06	17	9	5	3	4	240–250	178–202	162–168	138–144	0.82	1.30E-04	5.05E-02
06GH	5/23/06	33	7	8	5	5	238–252	176–200	160–168	136–150	0.94	1.43E-04	3.34E-02
06GOM	5/17/06	61	14	5	3	3	224–267	174–182	160–164	138–142	0.64	3.16E-03	1.05E-01
07SPA	5/09/07	31	8	9	5	4	238–265	176–202	162–170	138–144	0.97	8.76E-05	3.40E-02
07SPB	5/16/07	30	8	8	4	4	240–250	178–208	154–166	136–146	0.83	5.10E-04	5.17E-02
07SPC	5/23/07	32	7	6	6	7	238–250	176–202	160–172	132–148	0.81	3.14E-04	3.71E-02
07SPD	6/06/07	37	11	10	3	5	238–251	176–202	160–164	136–144	0.92	1.82E-04	3.85E-02
07GOM	5/30, 5/31/07	77	13	4	4	4	238–266	176–182	160–166	138–146	0.47	8.01E-03	1.17E-01
GOM combined		138	17	5	4	4	224–267	174–182	160–166	138–146	0.46	5.11E-03	1.09E-01
NMS combined		274	18	14	8	9	238–266	176–208	154–172	132–150	0.76	8.15E-05	2.70E-02

### Microsatellite analysis and ribotyping

For DNA extraction, mini-cultures were allowed to grow until each achieved a cell density of >100 cells per well. DNA was extracted from 200 μL of each culture using the Generation Capture Column Kit (Qiagen, Valencia, CA) following the manufacturers instructions, with an elution volume of 200 μL.

Of the microsatellite loci isolated by Nagai et al. (2004), 11 of 13 loci were tested with the small set of cultures from the GOM, after which the following four loci were selected for this study, based upon the number of alleles observed and amplification success (see Erdner et al. 2011): Atama 15, Atama 23, Atama 27, and Atama 39. PCR reactions contained ∼5 ng of template DNA, 0.2 mmol/L of each dNTP, 0.5 μmol/L of each designed primer pair, with one primer labeled with 6FAM, NED, PET, or VIC, 1× PCR buffer (10 mmol/L Tris-HCl, pH 8.3, 500 mmol/L KCl, 15 mmol/L MgCl_2_, 0.01% w/v gelatin), and 0.25 U of Ampli Taq Gold (Applied Biosytems Inc., Foster City, CA) to a total volume of 10 μL. The PCR cycling conditions were as follows: 10 min at 94°C, 38 cycles of 30 sec at 94°C, 30 sec at 60°C for the first 10 cycles and then at the primer-specific annealing temperature (Nagai et al. 2004) for the last 28 cycles, and 1 min at 72°C, and a final elongation for 5 min at 72°C. To determine if amplification was successful, PCR products were separated on a 1% TAE agarose gel adjacent to a 100 bp DNA ladder. For fragment analysis, PCR products were diluted 3–5× with nuclease-free water, and 1 μL of diluted product was mixed with 0.25 μL 500 LIZ Size Standard and 8.75 μL Hi-Di Formamide, and then analyzed using an ABI 3730xl DNA Analyzer (Applied Biosystems, Foster City, California). Allele sizes were determined using the program FPMiner (2005; BioinforSoft LLC, Beaverton, OR).

Numerous studies examining the global biogeographical distribution of the *A. tamarense* species complex have observed only the NA ribotype in the Northwestern Atlantic (Anderson et al. 1994, 2005b; Scholin et al. 1995; Lilly et al. 2007; Brosnahan et al. 2010; Crespo et al. 2011). However, to ascertain species identification of the populations examined in this study, a subset of the 11 sample sets representative of each water body and study year (06GOM, 06SPC, 07SPD) used in the microsatellite analysis were also amplified using PCR primers specific to the *A. fundyense* NA ribotype as described in Dyhrman et al. (2006).

### Microsatellite data analysis

Genetic diversity at each locus was measured by the number of alleles and gene (haplotype) diversity (Nei 1973) using POPGENE (1.32) (Ellstrand and Roose 1987; Yeh and Boyle 1997). Clonal diversity was calculated as the ratio of the number of unique four-locus genotypes (G) to the total number analyzed (N) (Ellstrand and Roose 1987). Additional tests to calculate the probability of identity (*P*_(ID)_ and *P*_(ID)sib_) for each locus and over all loci were performed using GIMLET v1.3.3 (Valière 2002) to assess the resolving power of the four microsatellite loci used in this study. For these tests, haploid genotype data were first converted to diploid format using the data conversion utility in GENEPOP (4.0) (Raymond and Rousset 1995; Rousset 2008). *P*_(ID)unbiased_ (herein referred to as *P*_(ID)_) provides a measure of the probability of observing identical multilocus genotypes between two individuals randomly sampled from a population, with a sample size correction. *P*_(ID)sib_ provides a more conservative measure and estimates match probabilities among sibs. According to guidelines provided by Waits et al. (2001), *P*_(ID)_ values between 0.01 and 0.0001 indicate sufficient resolving power by the microsatellite markers.

Pairwise genic comparisons using Fisher's exact test (G test) to assess genetic differentiation among samples, and tests for linkage disequilibrium were performed using GENEPOP (4.0) (Raymond and Rousset 1995; Rousset 2008) with Markov chain parameters of 2000 for dememorization number, 200 batches, and 2000 iterations per batch. Estimates of gene flow (*F*_ST_) were measured using Arlequin v3.5.1.2 (Excoffier et al. 2005). Bonferroni-corrected permutation tests were used to determine the significance of statistical values of pairwise comparisons.

Genetic structuring and admixture were further investigated using Bayesian cluster analysis as implemented in STRUCTURE 2.3.2 (Pritchard et al. 2000). The number of potential clusters (*K*) was first assessed using 30 runs performed at each *K* value from 1 to 10, each with a burn-in period of 10,000 steps and 10,000 Markov chain Monte Carlo repetitions. The log likelihoods of these data were used to calculate the statistic Δ*K*, to infer the uppermost level of population structure (Evanno et al. 2005). Simulations were performed using the admixture model with and without the locprior option, assuming correlated allele frequencies among populations. Clustering among samples was also inferred using principal components analysis (PCA) of *F*_ST_ values, performed using PCA-GEN (1.2) (Goudet 1999). Significance of each axis was determined using 10,000 randomizations of genotypes.

Finally, partitioning of genetic variance within and among samples was examined using analysis of molecular variance (AMOVA), with distance measures based on the number of different alleles, as implemented in Arlequin v3.5.1.2. Tests were first performed without groups, using: (1) the complete 2006–2007 GOM/NMS sample set; (2) 2006 NMS samples; and (3) 2007 NMS samples. Two additional tests were then performed in which the samples were organized into population groupings. The first test compared two groups: the GOM samples and the NMS samples. The second test included NMS samples only, and assessed interannual variation between 2006 NMS samples and 2007 NMS samples.

## Results

### Molecular identification of *A. fundyense* populations in the GOM and NMS

To ascertain that the genetic separation we observed among samples reflected population dynamics and not species dynamics, we assessed of a subset of samples used in the microsatellite analyses with primers specific to the NA ribotype of *A. fundyense*. PCR amplification of GOM and NMS sample subsets using these primers specific to the NA ribotype produced a single band of approximately 174 bp (Fig S1), confirming the species identification of these samples.

### Microsatellite data

The microsatellite loci employed in these studies were developed by Nagai et al. (2004) to examine *A. tamarense* strains from Japanese and Korean coastal waters (Nagai et al. 2007), and have also been employed to examine the genetic structure and dynamics of populations in the GOM (Erdner et al. 2011). A total of 412 isolates were genotyped for this study, 205 in 2006 and 207 in 2007 (Table 1). All loci examined were moderately or highly polymorphic. Atama15 and Atama23 displayed maximum numbers of 22 and 15 alleles, respectively, whereas maximum allele numbers displayed by Atama16 and Atama23 were lower, 8 and 9, respectively. A total of 27 alleles were restricted to samples from either the NMS or GOM; most of these alleles (24/27) were unique to samples collected from the NMS. Gene diversity (H_E_) at each allele was slightly lower than Nagai et al. (2004, 2007), but comparable to Erdner et al. (2011); with the exception of Atama39, allele numbers were higher in this study (Table S1). Linkage disequilibrium was not detected at any of the loci.

### Genetic diversity and marker resolution

Clonal diversity of bloom populations in the NMS, calculated as the proportion of unique four-locus genotypes (G:N), was remarkably high in both project years (Table 1), ranging from 81% to 97% genetically unique cells (mean = 88 ± 6%), and was highest in the early bloom samples (97% unique genotypes, 2006 and 2007). Clonal diversity was also comparable between the combined NMS yearly datasets from 2006 versus 2007 (83% and 86%, respectively; data not shown) and slightly lower in the total combined dataset (76%). However, clonal diversity was much lower in the GOM populations. Samples collected from Massachusetts Bay in 2006 and from Nantucket Shoals in 2007 comprised 64% and 47% unique genotypes, respectively, and clonal diversity of the combined GOM dataset was 46%.

Probability of identity (*P*_(ID)_) values of the NMS samples were within the guidelines suggested by Waits et al. (2001), indicating that the four microsatellite markers had sufficient resolving power for identifying individuals. However, values of the more conservative *P*_(ID)sib_ were slightly above the upper limit (Waits et al. 2001), ranging from 0.0272 to 0.0517 (combined dataset, 0.0272), thus limiting our ability to distinguish true clones. In contrast with the NMS samples, *P*_(ID)_ and *P*_(ID)sib_ values of samples from the GOM (combined dataset) were 0.0051 and 0.109, respectively, and were comparable between years (Table 1). These values were higher than those in the NMS, and exceeded the upper limit of 0.01, indicating that more loci would provide greater resolving power (also see Erdner et al. 2011).

### Genetic differentiation among populations

Pairwise comparisons using the Fisher's combined test of genotypic variation (Raymond and Rousset 1995) and Wright's *F*_ST_ estimator (Wright 1951) detected significant spatial and temporal genetic differentiation among *A. fundyense* blooms in different years, as well as between populations in the NMS and the GOM. In both project years, all populations sampled from the NMS were genetically distinct from those in the GOM; differentiation was statistically significant using both differentiation estimators, with *F*_ST_ values indicating moderate to high differentiation among the populations sampled (Table 2).

**Table 2:** Extent of genetic differentiation among *Alexandrium fundyense* populations from the Nauset Marsh System and the Gulf of Maine (GOM) sampled in 2006–2007. Differentiation estimators *F*_ST_ are shown in the lower half of the matrix and Fisher's combined test (*P* values) are shown in the upper half of the matrix. Bold numbers indicate significant values (*P* < 0.05) after sequential Bonferroni correction. H.S., highly significant

	Sampling date	06SPA	06SPB	06SPC	06SPD	06GHA	06GOM	07SPA	07SPB	07SPC	07SPD	07GOM
06SPA	4/26/06		0.1573	0.1207	0.0040	0.9749	**0.0000**	0.8228	0.3623	0.1185	0.3553	**0.0000**
06SPB	5/03/06	0.0349		0.9667	0.0013	0.2209	**H.S.**	0.0091	**0.0000**	0.3096	**0.0004**	**H.S.**
06SPC	5/10/06	0.0272	-0.0190		**0.0002**	0.1021	**H.S.**	0.0124	**0.0000**	0.0249	**0.0000**	**H.S.**
06SPD	5/17/06	**0.0970**	**0.0725**	**0.0842**		**0.0001**	**H.S.**	0.0030	**H.S.**	**0.0000**	0.0022	**H.S.**
06GHA	5/23/06	-0.0148	0.0226	0.0252	0.0831		**0.0000**	0.6618	0.0070	0.0491	0.5064	**0.0000**
06GOM	5/17/06	**0.0698**	**0.1369**	**0.1401**	**0.2984**	**0.0762**		**H.S.**	**0.0000**	**H.S.**	**H.S.**	0.2423
07SPA	5/09/07	-0.0084	0.0397	0.0388	**0.0902**	-0.0094	**0.0610**		0.0524	0.1887	0.1612	**H.S.**
07SPB	5/16/07	0.0040	**0.0864**	**0.0666**	**0.2029**	0.0218	**0.1065**	-0.0116		**0.0005**	**0.0009**	**0.0000**
07SPC	5/23/07	0.0102	-0.0346	-0.0251	**0.1408**	-0.0095	**0.0634**	-0.0279	**0.0735**		0.0095	**H.S.**
07SPD	6/06/07	0.0066	**0.0560**	**0.0662**	0.0758	-0.0095	**0.0845**	-0.0026	0.0258	-0.0048		**0.0000**
07GOM	5/30, 5/31/07	**0.0848**	**0.2045**	**0.2011**	**0.3442**	**0.1031**	-0.0016	**0.0816**	**0.1105**	**0.1677**	**0.1070**	

Within the NMS, we observed population differentiation among several samples collected from Salt Pond over the course of the bloom season in 2006 and again in 2007. In the 2006 dataset, *F*_ST_ values indicated significant genetic differentiation between the late bloom sample (06SPD), and those collected earlier in the bloom (Table 2). Results of the Fisher's combined test of genetic differentiation were slightly different, in that significant differentiation was only detected between 06SPC and 06SPD, and between 06SPD and 06GHA. These differences arise in part to the reduced statistical power associated with the Bonferroni-corrected *P*-value for the multiple pairwise comparisons (55) in the sample set (Bonferroni's adjustment, *P* = 0.00091). For example, when samples from 2006 were analyzed separately from the 2007 samples (10 pairwise comparisons), 06SPD was significantly different from all other early bloom populations (*P* = 0.005, data not shown). The genetic composition of the bloom in 2006 changed rapidly, as differences were detected between samples collected approximately 7 days apart. With the exception of 06SPD in the Fisher's test, the sample collected from Goose Hummock (06GHA), located in an area of the NMS known as Town Cove, was not significantly different from the samples collected in nearby Salt Pond.

Again in 2007, we observed temporal genetic differentiation among samples collected from Salt Pond (Table 2). Pairwise comparisons using Fisher's combined test indicated that the late bloom samples 07SPC and 07SPD were genetically distinct from 07SPB (*P* < 0.05), but not from 07SPA, which was collected during bloom initiation. However, comparisons using Wright's *F*_ST_ estimator revealed a slightly different pattern of genetic structure in which 07SPB was differentiated from 07SPC, but not from 07SPD. The *F*_ST_ value between 07SPB/07SPC was 0.074, indicating moderate genetic differentiation. In both analyses, the sample collected during bloom initiation, 07SPA, was not differentiated from samples collected subsequently during bloom development, or from the late bloom sample. As in 2006, changes in the genetic composition of *A. fundyense* populations collected from Salt Pond in 2007 occurred rapidly; again, on the order of 6–7 days.

In addition to temporal differentiation observed during the bloom, comparisons of the 2006 Salt Pond samples to those collected in 2007 revealed significant differences between sampling years. Both Wright's *F*_ST_ estimator and the Fisher's combined test revealed significant differences between 07SPB and 06SPB-06SPD (Table 2). Significant differences were also detected between the late bloom sample 07SPD and 06SPB-06SPC. Notably, early bloom samples from both 2006 and 2007 were largely undifferentiated from mid- and late-bloom samples, both within and between years.

Results of the AMOVA analysis indicated that the highest proportion of genetic variation (88–100%) was within samples, regardless of the population groupings examined (Table 3). In the overall dataset (GOM and NMS), among-group variation was highest when GOM and NMS samples were grouped (9.4%), reflecting the genetic separation among samples from these water bodies. Among-sample genetic variation was lower, but still significantly different when the NMS samples were examined separately from GOM samples (2.3%), but not when these samples were grouped according to study year (2006 vs. 2007). An AMOVA was also performed on NMS samples from each study year. In 2006, differences in genetic variation were significant among samples, but not in 2007 (Table 3).

**Table 3:** Analysis of molecular variance within and among samples from the Gulf of Maine (GOM) and Nauset Marsh System (NMS)

Analysis	df	SSD	Variance	Percent of variation
Without groups (all samples)				
Among samples	10	46.063	0.09346	7.45*
Within samples	402	466.588	1.16067	92.55
GOM versus NMS (two groups)				
Among groups	1	35.643	0.12328	9.37*
Among samples within groups	9	20.420	0.03201	2.43*
Within samples	402	466.588	1.16067	88.20*
Without groups (NMS samples only)				
Among samples	8	17.832	0.03039	2.28*
Within samples	266	346.829	1.30387	97.71
06NMS (five samples, no groups)				
Among samples	4	10.727	0.0454	3.18*
Within samples	140	193.280	1.38057	96.82
07NMS (four samples, no groups)				
Among samples	3	2.951	-0.00706	-0.59
Within samples	126	152.802	1.21272	100.59
06NMS versus 07NMS (two groups)				
Among groups	1	3.993	0.01449	1.08
Among samples within groups	7	13.839	0.02224	1.66*
Within samples	266	346.829	1.30387	97.26*

* P < 0.05.

**Figure 3:**
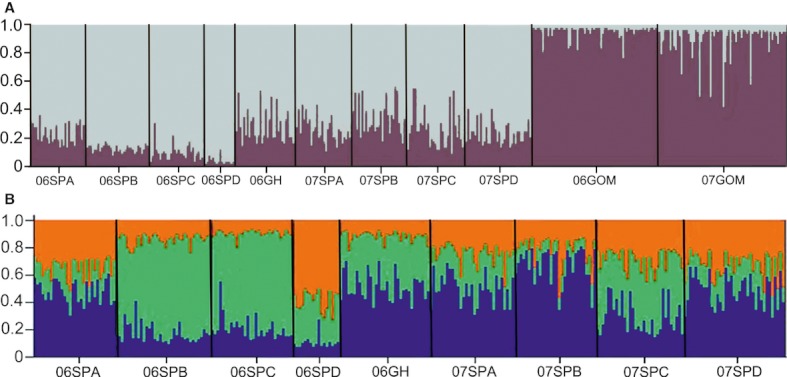
Population structure estimated by Bayesian cluster analysis implemented in STRUCTURE. Proportion of membership in each K-inferred cluster illustrated by the bar plot, and displayed underneath the sample names (A) GOM and NMS, 2006-2007, *K* = 2; (B) NMS (2006–2007), *K* = 3.

Relationships among samples were further examined using STRUCTURE analysis as well as PCA of pairwise *F*_ST_ values. STRUCTURE analysis plots of the Δ*K* statistic as a function of *K* showed maximum values at *K* = 2 for the entire GOM and NMS datasets. In these simulations, samples were divided into two clusters (Fig 3A), comprised of GOM and NMS samples, respectively. GOM samples were assigned to the “blue” cluster, with a proportional membership of >0.88. NMS samples were assigned to the “grey” cluster, and the proportional membership of these samples ranged from 0.66 (07SPB) to 0.96 (06SPD). In general, admixture was higher in the 2007 NMS samples than in the 2006 NMS samples. Examination of the NMS dataset separately showed maximum values of the Δ*K* statistic as a function of *K* at *K* = 3 (Fig 3B). In this analysis, all samples were admixed, although allele proportions are notably different among samples collected in 2006. The 06SPB and 06SPC samples had the highest proportional membership to the “green” cluster (>0.65), whereas 06SPD had the highest proportional membership to the “orange” cluster (>0.75). In the 2007 samples, differences in allele proportions were again observed. 07SPB had the highest proportional membership to the “blue” cluster (0.65) and similar to 06SPB and 06SPC, 07SPC had the highest proportional membership to the “green” cluster (0.42).

**Figure 4:**
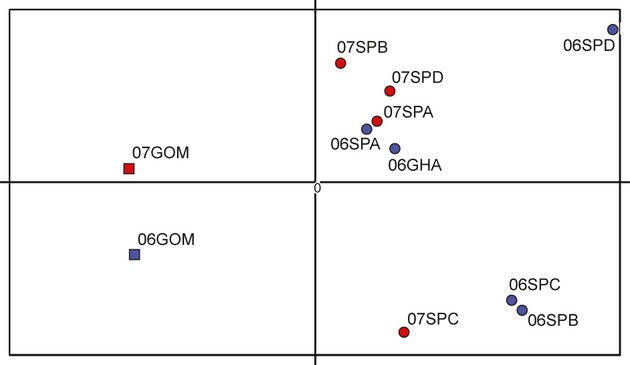
PCA of genetic differentiation among samples collected from the Nauset Marsh System (circles), and the Gulf of Maine (squares). Blue shapes represent samples collected in 2006, and red shapes represent samples collected in 2007.

The PCA separated the samples into groups (Fig 4): (1) 06GOM, 07GOM, (2) 06SPB, 06SPC, 07SPC, (3) 06SPD, and (4) the five remaining NMS samples. The first three axes of the PCA explained 63%, 15%, and 9% of the total variation in *F*_ST_, respectively. Variation was only significant along the first axis (*P* < 0.05), reflecting the separation of samples into two groups (GOM and NMS) in the STRUCTURE analysis. Subtle population structure was also apparent among the NMS samples. The grouping of 06SPB, 06SPC, 07SPC appeared to reflect the differentiation between these three samples and the remaining NMS samples, although differences detected in the pairwise comparisons were only statistically significant between this group and 07SPB and 07SPD (Fisher's exact test, *P* < 0.05). This group also shared the highest proportional membership to the “green” cluster in the STRUCTURE analysis (Fig 3B). Comparisons of genetic differentiation and STRUCTURE analysis also supported the separation of 06SPD from the other NMS samples observed in the PCA.

## Discussion

The four microsatellite loci employed in this study exhibited a high degree of diversity, and proved to be effective markers for detecting genetic differentiation in *A. fundyense* blooms in the NMS and GOM. These loci are a subset of 11 developed for *A. tamarense* in Japan (Nagai et al. 2004), which belong to the same *Alexandrium* clade as *A. fundyense* in the northeastern Atlantic (=North American/Group 1 ribotype). In this study, all four loci were polymorphic, and gene diversity was moderate or high at all loci, comparable to Nagai et al. (2004). Interestingly, differences in their resolving power were observed between the two water bodies, and were reflected in the lower *P*_(ID)_ values calculated for the NMS samples and higher levels of clonal diversity (Table 1).

Bloom populations in the NMS exhibited remarkably high levels of clonal diversity in both project years, ranging from 81% to 97% genetically unique cells (mean = 88 ± 6%). The discovery of such high levels of diversity in a geographically restricted area was unexpected, as the study area largely comprised a shallow, isolated salt pond <1000 m in diameter, and a nearby cove. In contrast with the NMS, diversity was lower in samples collected from the GOM: 64% in the 2006 sample collected from Massachusetts Bay and 47% in the 2007 sample collected from Nantucket Shoals. These levels are also lower than those documented previously by Erdner et al. (2011), which employed five loci to investigate GOM bloom populations in 2005. Clonal diversity of samples collected in 2005 ranged from 83% to 92%, thus providing further indication that four loci used in this study have less resolving power for the GOM populations.

One of the major objectives of this investigation was to determine the degree of connectivity between *A. fundyense* populations in the NMS and the GOM. Due to the restrictive nature of the Nauset inlet, which connects the terminal endpoints of the NMS to the open ocean through a complex maze of channels in the central marsh, *A. fundyense* in these endpoints appear to be contained within the estuary. Paralytic shellfish poisoning outbreaks in the NMS exhibit a pattern of localization and are generally confined to the terminal salt ponds and embayments, suggestive of “point sources” of cells originating from locally germinated cysts (Anderson et al. 1983; Anderson and Stolzenbach 1985). Paralytic shellfish poisoning on Cape Cod also occurs much earlier than in the GOM; within the NMS, Salt Pond is one of two key locations in which toxicity first appears, the second being Mill Pond, a terminal endpoint in the southernmost area of the system (Anderson et al. 1982; Crespo et al. 2011; Fig 2). These two locations feature the lowest flushing rates of the entire system, as well as the highest residence time of water, estimated to be between 5 and 10 days, with the longer values for deeper water (D. Ralston, pers. comm.).

The vertical migration behavior of *A. fundyense* has been shown to be an important mechanism limiting advective cell loss from Salt Pond, and may be equally as important as the hydrodynamic characteristics of this location in promoting the selective retention of *Alexandrium*. Studies of diel migration patterns by Anderson and Stolzenbach (1985) showed that *A. fundyense* exhibits surface-avoiding vertical migration behavior whereby the cells typically stop swimming upwards when they reach a light level of ∼150 μE m^−2^ sec^−1^, effectively separating them from the low salinity surface water lens that is flushed out of the salt pond with each ebb tide. This retention of cells would facilitate the local deposition of cysts that re-inoculate blooms in subsequent years (Anderson et al. 1983). Differences in the timing of blooms between these water bodies as well as the apparent retention of *A. fundyense* in the salt ponds of the NMS would prevent sexual reproduction between cells from the NMS and GOM, thus serving to reduce gene flow and reinforce the genetic differentiation between these populations.

We hypothesized that the localization of *A. fundyense* cells in Salt Pond from the combination of these behavioral and hydrographic factors restricts genetic exchange with GOM populations that may be present in the inlet channel and other well-mixed areas of the embayment.

This hypothesis was supported by data collected during both years of the study. In both 2006 and 2007, we observed moderate to high genetic differentiation between GOM and NMS populations (Table 2); AMOVA tests also showed the highest levels of among-group variation between populations from these bodies (Table 3). Two distinct clusters, comprising populations from each water body, were also apparent in the STRUCTURE analysis (Fig 3A). These data clearly indicate considerable genetic isolation between *A. fundyense* in the NMS and populations in the wider GOM, providing evidence that bloom dynamics and outbreaks of toxicity are indeed localized, driven by hydrographic and ecological factors specific to the embayment.

Within the NMS, we observed temporal genetic differentiation, both within and among sampling years. In both study years, successive sampling was timed to capture the genetic composition of populations at bloom initiation, development and progression, and during bloom decline; and in both 2006 and 2007, we observed temporal population differentiation. In 2006, the late-bloom sample 06SPD was significantly different from those collected in Salt Pond during bloom initiation and progression (Table 3; also evident in STRUCTURE analysis, Fig 3). Rapid differentiation was again observed in 2007; however, the population structure was more subtle. Although the Fisher's combined test showed significant genetic differentiation between each of the two late-bloom samples (07SPC, 07SPD) and 07SPB, these two late-bloom samples were not significantly different from each other, nor from the sample collected at bloom initiation (Table 2). Interannual comparisons of genetic differentiation and STRUCTURE analysis suggest that NMS bloom populations are comprised of at least three largely admixed subpopulations.

Considering the rapidity with which the genetic composition of populations in Salt Pond changed over the course of the bloom season, one explanation is mixing of genetically distinct cells from elsewhere in the NMS during the bloom. However, evidence regarding differences in the timing and population dynamics of the blooms among salt ponds in the NMS (Crespo et al. 2011), along with hydrodynamic data showing a long residence time of water within these salt ponds, do not support this hypothesis and instead suggest that blooms in the terminal endpoints of the system (e.g., Salt Pond, Mill Pond) are initiated locally and are largely retained within the endpoints of the system. Given the lack of information regarding the genetic composition of blooms from the remaining terminal endpoints in the NMS (e.g., Mill Pond), population mixing cannot be entirely ruled out, and is the subject of a system-wide investigation currently underway.

Other factors contributing to the genetic structure we observed include the effects of life-cycle transitions, such as differences in cyst maturation intervals and germination characteristics, which are important aspects of *Alexandrium* autoecology in Cape Cod salt ponds (Anderson et al. 1983; Anderson 1998). *Alexandrium* blooms appear earlier in these salt ponds than in the GOM and there are even differences among ponds in the NMS, in which blooms (and toxicity) are first observed in the its southern extreme (Mill Pond), followed by the northern extreme (Salt Pond), potentially due to differences in cyst abundance as well as physiological differences in cyst maturation intervals and timing of germination (Crespo et al. 2011). Studies of life-cycle dynamics in Salt Pond also revealed that the first planozygotes appear when populations as a whole were increasing rapidly, suggesting that differentiation and fusion of gametes occurred quite early in the stages of bloom development, well before the size of the bloom population peaks (Anderson et al. 1983). This process was remarkably consistent among populations from embayments on Cape Cod; in three different salt ponds, zygotes appeared approximately six divisions after the first vegetative cells were seen, suggesting endogenous or “clock”- related sexuality.

The initiation of sexuality in the early bloom population could curtail vegetative growth, leading to the formation of cysts and potentially to population decline. Differences in the timing of germination along with the early induction of sexuality may impede sexual recombination among physiologically (and genetically) distinct subpopulations that may be present in the terminal endpoints of the system, and those advected from the GOM, thus contributing to the clonal diversity and genetic divergence we observed. Anderson et al. (1983) posited that the encystment/excystment cycle temporally restricts the occurrence of the vegetative population and may not be optimized for rapid or sustained vegetative growth and bloom formation in shallow embayments. In this scenario, population succession could occur in which early germinating populations undergo vegetative growth, form gametes within a short period of time, and are then replaced by later germinating populations. This is supported by the 2006 data, which documented a genetically distinct late-bloom population. In 2007, however, population differentiation occurred earlier, and the late-bloom sample was not significantly different from the sample collected at bloom initiation. Thus, the relative importance of germination timing and early encystment relative to other factors influencing the population structure of blooms in Salt Pond still remains unclear.

A final consideration is that of grazers and parasites, which strongly influence the dynamics of *Alexandrium* in the NMS, and may also affect population structure. Watras et al. (1985) previously estimated grazing rates utilizing radioactive tracers (^14^C bicarbonate) and *A. fundyense* growth rates in Salt Pond, providing evidence that rates of grazing often exceeded growth rates, thus regulating the magnitude of the bloom and contributing to rapid bloom decline. These studies showed that historically, mortality due to zooplankton grazing has the potential to remove 100% of *Alexandrium* populations within a single day, and has been a key regulatory mechanism of population dynamics of *A. fundyense* in Salt Pond and other Cape Cod embayments (Turner and Anderson 1983; Watras et al. 1985). In addition to grazing, parasite-induced mortality by dinoflagellates in the genus *Amoebophrya* is a powerful top-down control of bloom-forming dinoflagellates in coastal environments, and has also been shown to drive annual cycles of dinoflagellate parasitoid successions observed in a coastal estuary in France (Chambouvet et al. 2008). A separate investigation conducted during the same period, as this study showed that *Amoebophrya* sp. parasitism is prevalent in Salt Pond, with estimates of host infection rates as high as 90% (Sengco and Coats, unpubl. ms.). Thus, there is historical and current evidence that both grazing pressure and parasitism can strongly influence *Alexandrium* dynamics in Salt Pond, and therefore may contribute to the population structure. Microbial food web models incorporating parasitism by *Amoebrophrya* spp. can result in the near extirpation of dinoflagellate blooms (Montagnes et al. 2008), thus influencing the genetic dynamics of the successive populations rebounding from infection. Such strong selective pressure may consequently help maintain genotypic diversity in the population.

The results described herein clearly highlight the need for further investigation of the roles of population mixing and selective forces in the genetic dynamics of bloom populations in the NMS. To begin addressing these factors, an expanded investigation is underway of the genetic composition of *A. fundyense* populations from the other terminal endpoints of the NMS. Continued studies of the population and genetic dynamics of *A. fundyense* in the NMS, coupled with future investigations of host–parasite interactions and grazing pressure, will provide a better understanding of the evolutionary ecology of blooms and potential role of top-down controls in driving genetic dynamics. Due to their small geographic scale and limited connectivity, NMS salt ponds provide a novel system for investigating regulators of bloom development and termination, such as life-cycle events, as well as the influence of selective forces on population dynamics, all of which are otherwise difficult or impossible to study in the adjacent open-coastal waters or larger estuaries.

## Conclusions

This study examined *A. fundyense* populations within a shallow embayment on Cape Cod, MA, to investigate the genetic characteristics of localized blooms and assess their connectivity with regional GOM populations. Comparisons of genetic differentiation showed that *A. fundyense* blooms in the NMS were genetically distinct from populations in the GOM, indicating that the localization of *A. fundyense* populations by behavioral and hydrographic factors effectively restricts genetic exchange with GOM populations. *A. fundyense* blooms in the NMS were characterized by extensive genetic diversity and rapid temporal genetic differentiation. On the basis of preliminary data from other investigations, we hypothesize that the genetic dynamics observed in Salt Pond may be influenced by one or more of the following factors: (1) life-cycle stages, including early onset of sexuality, and differences in cyst maturation intervals and timing of germination; (2) strong selection pressure exerted by *Amoebophrya* sp. parasitism and potentially from zooplankton grazing; and, (3) the mixing of distinct genotypes from a separated population elsewhere in the NMS.

## Footnotes

1 Two morphotypes, *A. tamarense* and *A. fundyense*, occur in the GOM region. However, throughout the long history of research (e.g., Anderson et al. 1994, 2005a; Scholin et al. 1995; Lilly et al. 2007; Brosnahan et al. 2010; Crespo et al. 2011), only one ribotype (i.e., North American [NA] ribotype) of the *Alexandrium tamarense* species complex has been observed throughout the GOM and associated embayments. Both morphotypes are thus considered to be varieties of the same species. Accordingly, for the purpose of this study, the name *A. fundyense* is used to refer to both forms.

## Supporting Information

**Table S1.** Characteristics of the microsatellite loci used to assess genetic diversity of *Alexandrium fundyense* populations from the GOM and the NMS on Cape Cod, MA, USA. Results of this are compared with those observed by Nagai et al. (2004, 20074) in Japanese toxic *A. tamarense*. *N*, individuals, *N*_a_, maximum number of alleles, R-size ranges of the alleles, H_**E**_, gene diversity, ND, no data.

**Figure S1**. PCR products from (A) 06GOM, (B) 06SPC, and (C) 07SPD. Amplification performed using PCR primers specific to the NA ribotype of *Alexandrium fundyense*.
