# Supplementary material for: Extensive genetic diversity and rapid population differentiation during blooms of Alexandrium fundyense (Dinophyceae) in an isolated salt pond on Cape Cod, MA, USA
Source: Ecol Evol. 2012 Sep 13;2(10):2588–99. doi: 10.1002/ece3.373 (PMC3492784; doi:10.1002/ece3.373)
Supplement: Supplementary file 1 [file ece30002-2588-SD1.docx]

Supplementary Table 1. Characteristics of the microsatellite loci used to assess genetic diversity of *A. fundyense* populations from the GOM and the NMS on Cape Cod, MA, USA. Results of this are compared with those observed by Nagai *et al*. (2004, 2007) in Japanese toxic *A. tamarense*. N=individuals, N_a_ = maximum number of alleles, R-size ranges of the alleles, H**_E_** = gene diversity, ND=no data.

| **Locus** | This study | Nagai *et al.* (2004) | Nagai *et al.* (2007) |
| --- | --- | --- | --- |
| **Atama 15** |  |  |  |
| **N** | 408 | 18 | 491 |
| **N_a_** | 22 | 10 | 15 |
| **R** | 224-267 | 238-259 | 232-270 |
| **H_E_** | 0.751 | 0.904 | 0.90* |
| **Atama 23** |  |  |  |
| **N** | 372 | 19 | 474 |
| **N_a_** | 15 | 5 | 9 |
| **R** | 174-208 | 175-182 | 172-202 |
| **H_E_** | 0.783 | 0.789 | 0.80* |
| **Atama 27** |  |  |  |
| **N** | 389 | 15 | ND |
| **N_a_** | 8 | 6 | ND |
| **R** | 154-172 | 155-177 | ND |
| **H_E_** | 0.588 | 0.889 | ND |
| **Atama 39** |  |  |  |
| **N** | 411 | 20 | 485 |
| **N_a_** | 9 | 6 | 11 |
| **R** | 132-150 | 138-150 | 136-162 |
| **H_E_** | 0.627 | 0.779 | 0.80* |

*maximum value reported for the populations analyzed.
