# Supplementary figures and images for: Extensive genetic diversity and rapid population differentiation during blooms of Alexandrium fundyense (Dinophyceae) in an isolated salt pond on Cape Cod, MA, USA
Source: Ecol Evol. 2012 Sep 13;2(10):2588–99. doi: 10.1002/ece3.373 (PMC3492784; doi:10.1002/ece3.373)

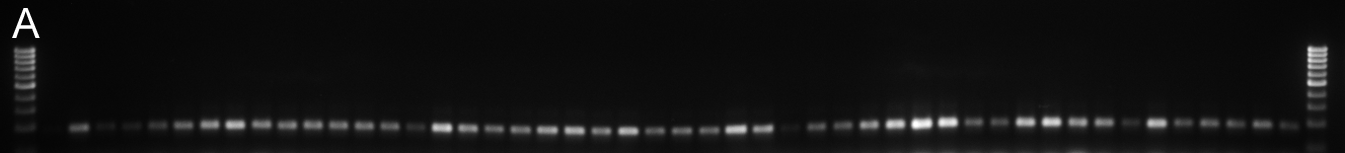

Supplement: Supplementary file 2 [file ece30002-2588-SD2.tif]

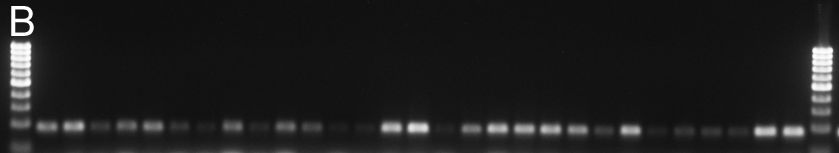

Supplement: Supplementary file 3 [file ece30002-2588-SD3.tif]

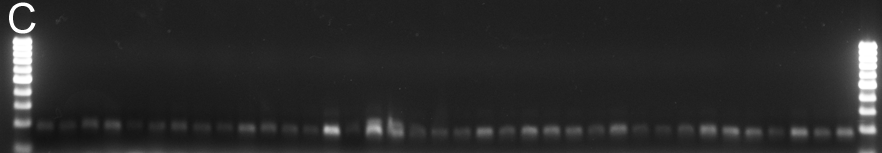

Supplement: Supplementary file 4 [file ece30002-2588-SD4.tif]
